# Effects of Ferrocene and Ferrocenium on MCF-7 Breast Cancer Cells and Interconnection with Regulated Cell Death Pathways

**DOI:** 10.3390/molecules28186469

**Published:** 2023-09-06

**Authors:** Cristina Favaron, Elisabetta Gabano, Ilaria Zanellato, Ludovica Gaiaschi, Claudio Casali, Maria Grazia Bottone, Mauro Ravera

**Affiliations:** 1 Department of Biology and Biotechnology “L. Spallanzani”, University of Pavia, Via Ferrata 9, 27100 Pavia, Italy; cristina.favaron01@universitadipavia.it (C.F.); ludovica.gaiaschi01@universitadipavia.it (L.G.); claudio.casali01@universitadipavia.it (C.C.); 2 Department of Sustainable Development and Ecological Transition, University of Piemonte Orientale, Piazza S. Eusebio 5, 13100 Vercelli, Italy; elisabetta.gabano@uniupo.it; 3 Department of Sciences and Technological Innovation, University of Piemonte Orientale, Viale Teresa Michel 11, 15121 Alessandria, Italy

**Keywords:** organoiron derivatives, regulated cell death, ferroptosis, oxidative stress

## Abstract

The effects of ferrocene (**Fc**) and ferrocenium (**Fc^+^**) induced in triple negative human breast cancer MCF-7 cells were explored by immunofluorescence, flow cytometry, and transmission electron microscopy analysis. The different abilities of **Fc** and **Fc^+^** to produce reactive oxygen species and induce oxidative stress were clearly observed by activating apoptosis and morphological changes after treatment, but also after tests performed on the model organism *D. discoideum*, particularly in the case of **Fc^+^**. The induction of ferroptosis, an iron-dependent form of regulated cell death driven by an overload of lipid peroxides in cellular membranes, occurred after 2 h of treatment with **Fc^+^** but not **Fc**. However, the more stable **Fc** showed its effects by activating necroptosis after a longer-lasting treatment. The differences observed in terms of cell death mechanisms and timing may be due to rapid interconversion between the two oxidative forms of internalized iron species (from Fe^2+^ to Fe^3+^ and vice versa). Potential limitations include the fact that iron metabolism and mitophagy have not been investigated. However, the ability of both **Fc** and **Fc^+^** to trigger different and interregulated types of cell death makes them suitable to potentially overcome the shortcomings of traditional apoptosis-mediated anticancer therapies.

## 1. Introduction

**Figure 1:**
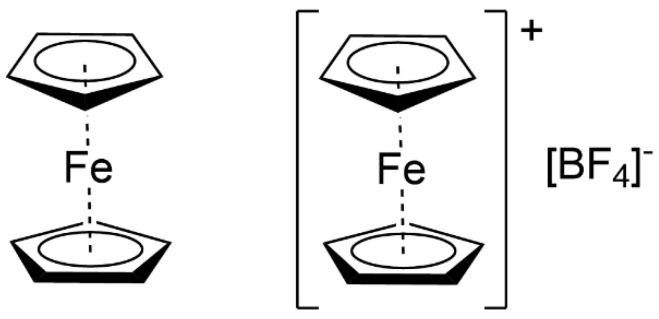
Sketch of ferrocene (**Fc**) and ferrocenium tetrafluoroborate ([**Fc^+^**][BF_4_]^−^).

In 2023, a paper dealing with ferrocene (bis(η^5^-cyclopentadienyl) iron, [Fe(η^5^-C_5_H_5_)_2_], **Fc**) and its oxidized form ferrocenium (bis(η^5^-cyclopentadienyl) iron(1+), [Fe(η^5^-C_5_H_5_)_2_]^+^, **Fc^+^**) (Figure 1) may seem anachronistic. According to “Nomenclature of Inorganic Chemistry—IUPAC Recommendations 2005”, the name ‘*bis(η^5^-cyclopentadienyl)iron(1+) is strongly preferred to ferrocenium for [Fe(η^5^-C_5_H_5_)_2_]^+^*’. However, for simplicity and by analogy with ferrocene, ferrocenium and **Fc^+^** will be used throughout the paper.

Since their discovery in the 1950s [1,2], **Fc** and its derivatives have attracted much attention due to their high chemical stability and well-known synthetic chemistry, making them easy to manipulate to obtain customized characteristics [3]. Furthermore, the unique redox properties of **Fc** and **Fc^+^** have been successfully exploited for the development of sensors and supramolecular switches [4]. Finally, **Fc** derivatives have been used in a wide range of material science applications [5]; it is hard to find a field where they have not played a role. Even today, **Fc** is still a subject of interest. From a quick search on the Web of Science^TM^, the number of articles citing the word ‘ferrocen*’ as one of the ‘Topics’ was 400 in 1991; this number increased to 1052 in 2007, maintaining a value > 1000 up to 2021 and decreasing to 969 in 2022.

Quite naturally, biological and medicinal chemistry, as well as pharmacology, have benefitted from the peculiar characteristics of **Fc**. Ferrocene behaves in many aspects as an aromatic, electron-rich, organic compound and is activated toward electrophilic reactions almost like phenol. In 1996, Jaouen et al. replaced the phenyl group of hydroxytamoxifen, the active metabolite of tamoxifen (a hormone therapy drug used to treat estrogen receptor-positive breast cancer). The resulting ‘hydroxyferrocifens’ were designed to combine the anti-estrogenic effect of tamoxifen with the potentially cytotoxic properties of ferrocene. In 1997, Brocard et al. modified the standard antimalarial drug chloroquine, introducing a **Fc** moiety. The resulting compound, ferroquine, is active against chloroquine-sensitive *Plasmodium* parasites but also against chloroquine-resistant strains [6,7,8,9,10,11,12,13]. These two classes of compounds represent some of the spearheads of ‘bioorganometallic chemistry’. This is a well-established area of research which extends organometallic chemistry into biology (covering enzymes and cofactors that are organometallics or use organometallic chemistry) and medicine (dealing with medicinal applications or toxicological aspects of organometallic compounds) [14,15].

The first observation of the potential antineoplastic activity of **Fc** salts dates back to the 1980s. In 1984, Köpf-Maier and Neuse provided the first evidence on the antineoplastic activity of some **Fc^+^** salts against an Ehrlich ascites tumor (EAT) in mice [16,17]. Some derivatives induced cure rates of 70–100% over a broad dose range. Furthermore, other tumor cell lines were inhibited by **Fc^+^** compounds, such as B16 melanoma, colon 38, and Lewis lung carcinomas. Importantly, water-insoluble **Fc** did not show identifiable tumor inhibiting activity. These findings demonstrate that the lipophilicity of the neutral organometallic compound should guarantee a high cell uptake; water solubility is of paramount importance to allow correct distribution of the molecule within the body [16,18,19]. Subsequently, Neuse and Kanzawa observed the metabolic oxidation of **Fc** derivatives from in vitro clonogenic assays [20].

Over the years, many studies have suggested that the antiproliferative activity of compounds containing ferrocene is mainly due to the intracellular generation of reactive oxygen species (ROS; e.g., singlet oxygen ^1^O_2_, superoxide anion radicals O_2_^•−^, hydroxyl free radicals HO^•^, hydrogen peroxide H_2_O_2_, among others [21]). The relationship between **Fc** derivatives and ROS was first demonstrated in 1997. Tamura et al. reported that **Fc** derivatives could influence DNA cleavage after the generation of HO^•^ radicals [22], whose production was later confirmed with ESR measurements by Osella et al. [23,24].

ROS are naturally generated by cells as an inevitable consequence of metabolism, but also as a result of pathogenic mechanisms, including neoplastic transformation [25]. The hyperproliferation of tumors is accompanied by a high production of ROS; cancer cells shape their metabolisms to survive under those stressful conditions to sustain an even more uncontrolled proliferation. However, at the same time, tumor cells must keep ROS production below the thresholds that trigger several types of programmed cell death. This feature makes cancer cells more sensitive to a further increment in ROS so that overwhelming their redox adaptation could induce an oxidative stress incompatible with their life [26]. For some clinically approved anticancer drugs, the increase in intracellular ROS has been recognized, at least as a side mechanism of action. As an example, elevated levels of oxidants in the circulation were reported after the administration of epirubicin and doxorubicin to cancer patients. Both drugs generate ROS that lead to DNA damage and, subsequently, antitumor activity [27,28]. Furthermore, vinorelbine, which belongs to the class of vinca alkaloids, has been shown to deplete antioxidant intracellular glutathione (GSH) and increase intracellular ROS production [29].

Therefore, the modulation of oxidative stress appears to be an attractive and promising strategy for cancer therapy [30,31,32]. Unlike purely organic molecules, coordination and organometallic compounds can be particularly suitable for this approach because the metal center can be easily involved in redox reactions under biological conditions. The switch between different oxidation numbers can induce the direct or indirect formation of ROS [33,34].

As previously stated, **Fc^+^** derivatives offered better in vivo performances than those of **Fc**, possibly because of their better water solubility and, hence, bioavailability. Indeed, **Fc** derivatives bearing different functionalities on the cyclopentadienyl rings may not be completely inactive because of an increased water solubility or decreased oxidation potential. Furthermore, the shell of the cyclopentadienyl ligands stabilizes and preserves the redox center by extracellular processes and makes the overall complexes lipophilic enough to easily enter cells. Finally, once in a cellular environment, the compound could exist as a **Fc^+^**/**Fc** couple; the equilibrium position depends on the redox conditions of the biological compartment [16,17,22,23,24,35].

In its lower oxidation form, **Fc** can react with endogenous H_2_O_2_ (Fenton-type reaction), while the corresponding oxidized ions **Fc^+^** can, in turn, be reduced by superoxide anion (Haber–Weiss cycle) or GSH. Therefore, the intracellular production of HO^•^ could potentially be driven by both complexes, regardless of the initial oxidation state of the metal core. The highly toxic hydroxyl radical HO^•^ is able to react at a nearly diffusion-limited rate with several cellular components, thus causing cells to die.

However, up to now it has always been implicitly considered that the induction of cell death is a consequence of the direct oxidative damage caused by ROS in cellular components. Today, ROS are also recognized to have roles in the regulation of many aspects of cell function. Specific ROS can target and modify the function, localization, and/or activity of many different proteins, controlling cell functions including cell death [36,37].

There are several different ways for a cell to die [38]. Knowledge of these mechanisms is not only of fundamental physiological significance but also of pathological importance. For instance, changes in cell death mechanisms are common in cancer and can lead to drug resistance, and therefore to treatment failures. In addition, the reactivation of modified or silenced cell death pathways can provide more effective cancer treatment strategies [39].

Ferroptosis is an iron-dependent regulated cell death (RCD), reported for the first time in 2012, characterized by excessive ROS-induced lipid peroxidation [40]. Compared to non-malignant cells, the increased metabolic activity of tumor cells requires a strong demand for iron. Excess iron in cells can switch on Fenton chemistry, directly generating ROS or activating iron-containing enzymes that promote lipid peroxidation. For this reason, in contrast to autophagy, apoptosis, and other types of cell death, ferroptosis is characterized by peculiar cytological alterations. These are caused by the loss of permeability of the cell membrane, which occurs due to severe lipid peroxidation reactions [41].

Studies have found that ferroptosis is more commonly observed in cancer cell lines than in normal cell lines, although different cancer cells show variable sensitivities [42,43]. Importantly, due to its non-apoptotic nature, ferroptosis could overcome the shortcomings of traditional therapies mediated by the apoptotic pathway. Therefore, the induction of ferroptosis could open new horizons for cancer chemotherapy.

In the last three years, **Fc** and its derivatives have been incorporated into several nanomaterials for oncotherapy to boost their performance, also with the induction of ferroptosis. Furthermore, the possibility of the selective production of ROS makes ferrocene-containing agents suitable for chemodynamic therapy that uses Fenton or Fenton-like reactions to convert endogenous H_2_O_2_ to highly toxic HO^•^, which in turn can induce tumor apoptosis [44,45,46,47].

In the present study, the effects of **Fc** and **Fc^+^** induced on the life and death of MCF-7 breast cancer cells will be explored. In particular, the main aim was to obtain further information on the induction of ferroptosis, which has only been marginally associated with ferrocene derivatives.

However, even if ferroptosis is an independent type of RCD, it shares a common pathway with other types of intracellular death programs. For this reason, this study will start with the effects of **Fc** and **Fc^+^** on the cell cycle, apoptosis and necroptosis, as well as on the evaluation of the essential regulators of ferroptosis (ROS) and their progenitor (iron uptake in model cells and stability of **Fc^+^** in model solutions). Finally, the morphological changes in the treated cells, analyzed by transmission electron microscopy, will provide a timeline for the activation of different RCD mechanisms.

This multidisciplinary study aims to provide new information on the effects of **Fc** and **Fc^+^** on cancer cells and their ability to trigger multiple and interregulated pathways. In this framework, the (oldies but goodies) ferrocene derivatives could represent an element inside the formulation of an intra- or intermolecular anticancer combination therapy.

## 2. Results and Discussion

### 2.1. Cell Cycle Analysis

**Figure 2:**
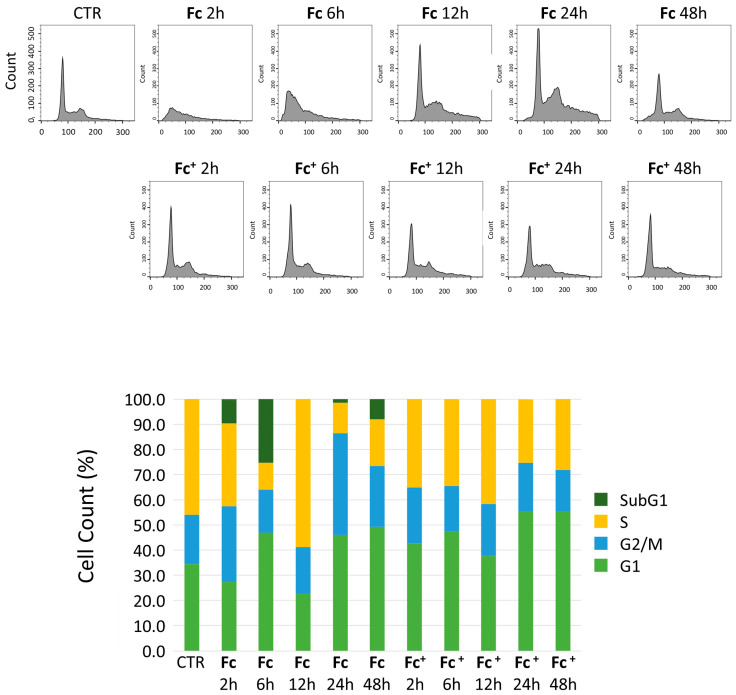
(**Top**) Cytofluorimetric analysis. Graphs showing DNA content after PI staining on human MCF-7 cells in control condition and after 2, 6, 12, 24, and 48 h of CT with **Fc** and **Fc^+^** (200 μM). (**Bottom**) Histograms showing the distribution of cells across the different cell cycle phases under control conditions and after **Fc** and **Fc^+^** treatments.

To investigate the effects on the cell cycle of the compounds tested, triple negative human breast cancer cells MCF-7 were treated with 200 μM concentrations of **Fc** and **Fc^+^**, respectively. At different time points, DNA was stained with propidium iodide (PI) and analyzed by flow cytometry. As shown in Figure 2, under control conditions, the recorded events were well distributed between the four different phases of the cell cycle (i.e., G1, S, G2, and M). On the other hand, after 2 and 6 h of continuous treatment (CT) with **Fc**, the peaks were no longer identifiable, indicating a high presence of cell death. However, after 12, 24, and 48 h, the analysis showed a different distribution of cells across the different phases of the cell cycle with a slightly prominent G2/M peak after 24 h of CT with **Fc**, indicating a possible blockage of the cell cycle in this phase. Notably, a small sub-G1 peak was clearly visible after 24 and 48 h of CT with **Fc**.

In contrast, in samples treated with **Fc**^+^, an accumulation of cells was detected in the G1 phase at all time points tested, especially 24 and 48 h CT.

Treatment with **Fc** has a more direct impact on the cell cycle. Flow cytometry data show that **Fc** efficiently causes an accumulation of cells in the G2/M phase after 24 h of exposure and a decrease in G1 and proliferating cells, suggesting a late blockage of cell proliferation. Furthermore, the presence of a sub-G1 peak revealed the activation of cell death after a longer exposure. In contrast, **Fc^+^**-treated samples showed a G1 accumulation, which is more prominent after 24 and 48 h, suggesting that **Fc^+^** mainly affects the cell cycle after longer exposure.

### 2.2. **Fc** and **Fc^+^** Impaired Oxidative Stress Response

To confirm and evaluate the oxidative stress response induced by the compounds under investigation, the expression of COXIV and nitrotyrosine after treatment with **Fc** and **Fc^+^** was investigated.

COXIV is the largest nuclear-coded subunit of the Cytochrome C Oxidase (COX) enzyme, a major regulator of mitochondrial ROS homeostasis [48]. A deficiency in COXIV has been shown to reduce COX activity and mitochondrial function and enhance ROS accumulation [49].

Nitrotyrosine is a relatively stable product formed through different reaction pathways, for example, during the reaction of peroxynitrite with tyrosine. For this reason, nitrotyrosine has been identified as an indicator of cell damage and inflammation; measuring its concentration will serve as a marker for damage caused by reactive nitrogen species (RNS), such as NO, in the cell [50].

**Figure 3:**
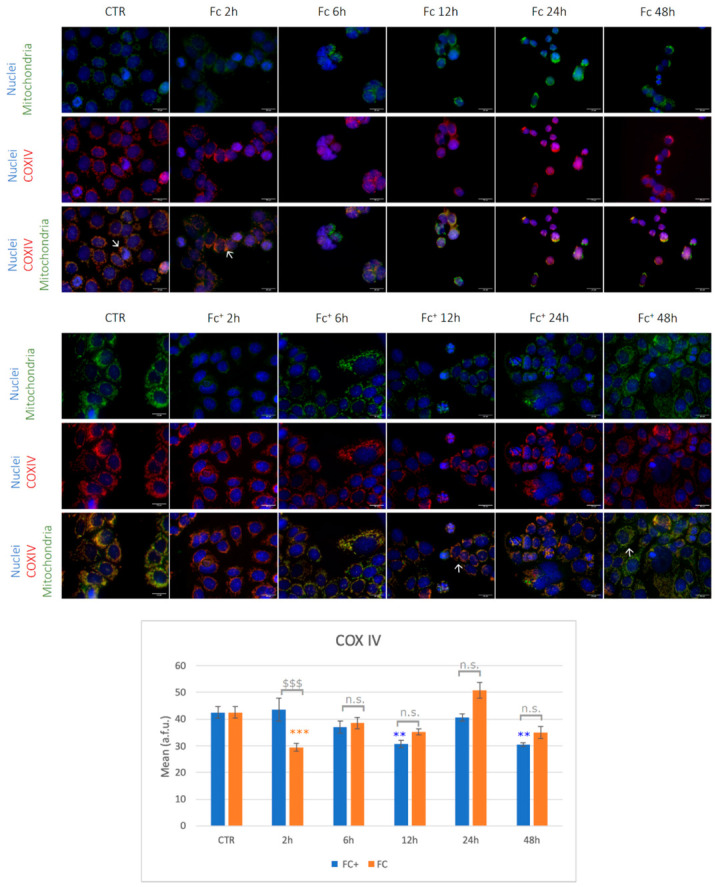
(**Top**) Double immunocytochemical reaction for mitochondria (green fluorescence) and COXIV (red fluorescence); DNA counterstaining with Hoechst 33258 (blue fluorescence) in control condition and samples treated with **Fc** and **Fc^+^** (200 μM). Bar = 20 μm; magnification: 60×. See the main text for the meaning of the arrows. (**Bottom**) The histogram represents the relative expression of COXIV. Statistical analysis (one-way Anova): *p* < 0.0001. Statistical significance between * control condition and treated samples; statistical significance between $ **Fc**- and **Fc^+^**-treated samples (two-tailed unpaired *t*-test); *p*-values: (**) *p* < 0.01; ($$$), (***) *p* < 0.005; n.s. = not significant.

As shown in Figure 3, COXIV and mitochondrial signals co-localized in controls and treated samples as expected (indicated by arrow). However, while in untreated cells mitochondria appeared with their characteristic spotted-like shape, in **Fc**-treated samples they show homogeneous fluorescence morphologies with a tendency to form clusters (arrows). Taking into account COXIV expression, a significantly lower signal was detected compared to the control condition after 2 h of CT with **Fc**. In the other tested time points, fluctuations of the signal were observed, although without statistically significant changes vs. control. Moving to the samples treated with **Fc^+^**, mitochondria appeared with altered elongated morphology, as indicated by arrows, while COXIV was generally down-regulated, even if statistical significance was reached only after 12 and 48 h of CT vs. controls (Note: all statistical results of the immunofluorescence experiments are shown in Table S1, Supplementary Material).

**Figure 4:**
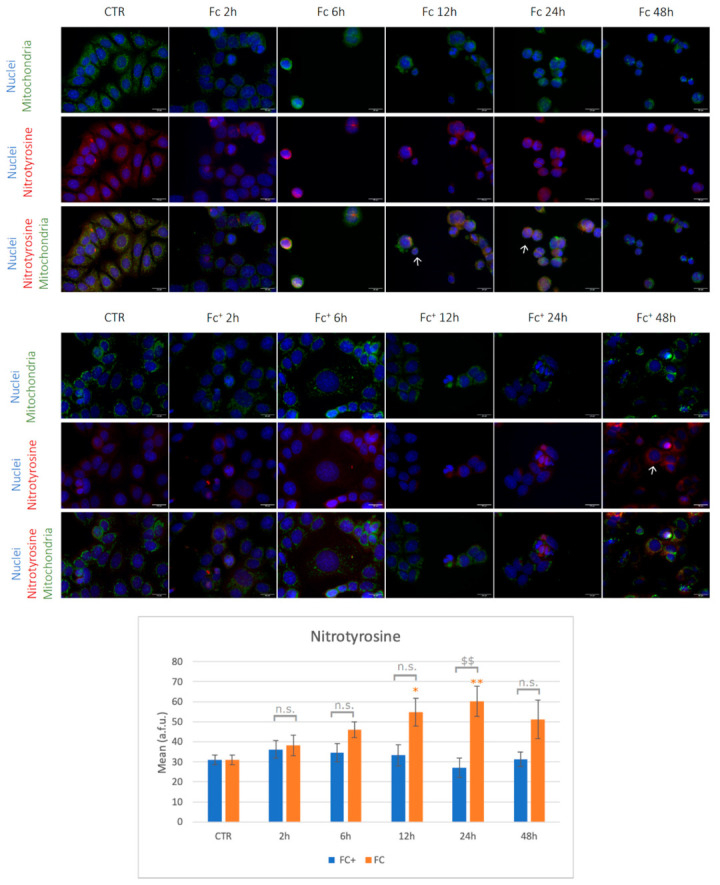
(**Top**) Double immunocytochemical reaction for mitochondria (green fluorescence) and nitrotyrosine (red fluorescence); DNA counterstaining with Hoechst 33258 (blue fluorescence) under control condition and treated samples with **Fc** and **Fc^+^** (200 μM). Bar = 20 μm; magnification: 60×. See the main text for the meaning of the arrows. (**Bottom**) The histogram represents the relative expression of nitrotyrosine. Statistical analysis (one-way Anova): *p* = 0.0002. Statistical significance between * control condition and treated samples; statistical significance between $ **Fc**- and **Fc^+^**-treated samples (two-tailed unpaired *t*-test); *p*-values: (*) *p* < 0.05; ($$), (**) *p* < 0.01; n.s. = not significant.

Nitrotyrosine experiments are reported in Figure 4 (see also Table S1, Supplementary Material). In untreated samples, cells showed regular shaped mitochondria well distributed around the nucleus. After treatment with **Fc,** cells appeared smaller, round-shaped, and with clustered mitochondria, as previously described (indicated by arrows). After 12 and 24 h of exposure to **Fc,** the anti-nitrotyrosine antibody was significantly higher compared to the control condition. On the other hand, cells treated with **Fc^+^** show altered mitochondria, but nitrotyrosine levels were not significantly affected by the pharmacological insult. Overall, the lower levels of COXIV in **Fc** and **Fc^+^** point to mitochondrial dysfunction as a consequence of ROS accumulation, as expected, which occurs earlier after **Fc** treatments compared to **Fc^+^**. Furthermore, a faster accumulation of ROS led to RNS production in **Fc** after 12 and 24 h. However, in **Fc^+^**-treated samples, lower levels of COXIV were detected after longer exposures, suggesting delayed accumulation of ROS compared to **Fc**. Furthermore, given the delayed accumulation of ROS, no significant increments in nitrotyrosine levels were detected between the time points tested here, compared to the control conditions [51].

### 2.3. Effect of **Fc** and **Fc^+^** on the ROS Production by Using the D. discoideum Model Organism

To deepen the knowledge on the oxidative stress caused by **Fc** and **Fc^+^**, one can take advantage of the use of *Dictyostelium discoideum* (*D. discoideum*, nicknamed dicty afterward). This social amoeba is a professional phagocyte and pathogen host that has long been recognized for its value as a biomedical and ecotoxicological model organism. In fact, its cellular structure and ease of cultivation makes it a simple but excellent experimental model for studying various signal transmission pathways [52,53]. In particular, dicty is known to be unusually resistant to DNA-damaging agents (UV light, γ radiation, drugs, etc.) and oxidative stress [54]. Moreover, the development of dicty occurs under starving conditions in simple salt solutions or even in water and it is not necessary to add divalent transition metals [55]. Therefore, cellular accumulation data can be obtained unambiguously using an atomic absorption/emission technique without employing radioactive derivatives of ^57^Fe as for human cells. Importantly, dicty is also one of eight non-mammalian model organisms recognized by the National Institute of Health (NIH) in the United States for its utility in the study of fundamental molecular processes of human medical importance [56].

**Figure 5:**
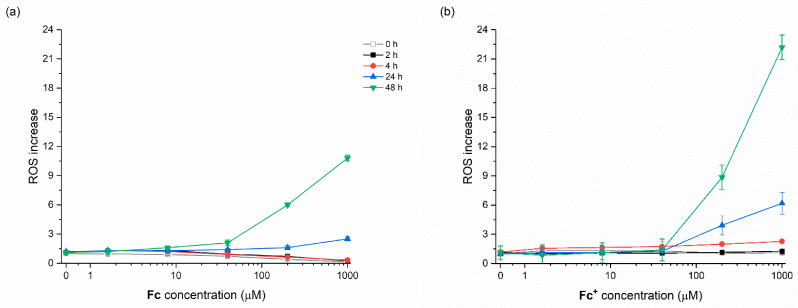
ROS increase induced by increasing concentrations of (**a**) **Fc** and (**b**) **Fc^+^** measured by the dichlorofluorescein (DCF) assay. After 2, 4, 24, and 48 h of continuous treatment, dicty cells were washed and loaded with 2′,7′-Dichlorodihydrofluorescein diacetate (H_2_-DCF-DA) (15 min), then washed and read.

A 2′,7′-Dichlorodihydrofluorescein diacetate (H_2_DCF-DA) was used as a fluorescent indicator of ROS formation. H_2_DCF-DA can penetrate the cell membrane and once inside the cell, intracellular esterase enzymes will cleave the acetate groups and trap the 2′,7′-dichlorodihydrofluorescein (H_2_DCF) within the cell. In the presence of ROS, H_2_DCF is rapidly oxidized to dichlorofluorescein (DCF), which is highly fluorescent. Because the dye does not affect cell viability, ROS formation can be tracked in real time by making periodic fluorescent measurements of cells with a fluorimeter. Figure 5 shows the ROS formation induced by increasing **Fc** and **Fc^+^**, particularly after 24 and 48 h CT.

**Figure 6:**
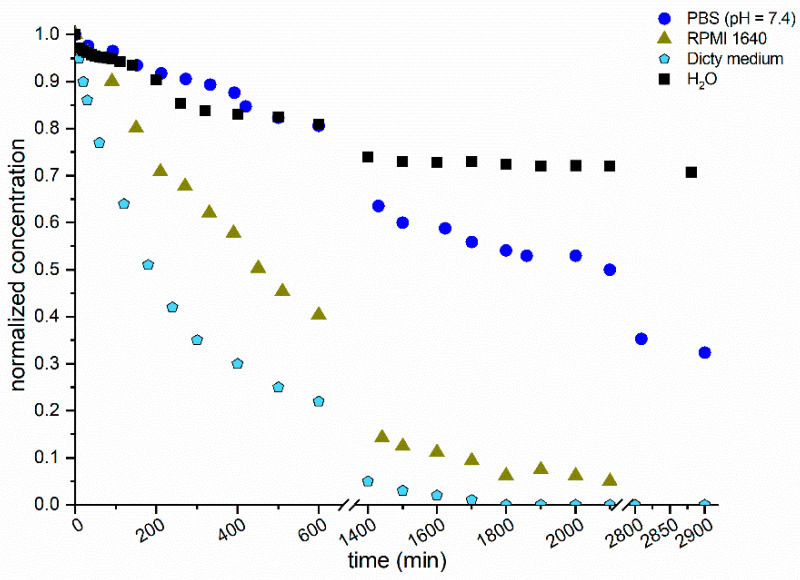
Stability in solution vs. aging time in different model solutions (H_2_O; phosphate-buffered saline, PBS, pH = 7.4; RPMI 1460 and dicty medium) at 25 °C. Data were obtained by using cyclic voltammetry (peak current, *i*_p_) or UV-vis spectroscopy (absorption A at λ_max_ around 618 nm, depending on the solution). To compare the results, concentrations were normalized against the *i*_p_ or A values at time zero.

To evaluate the stability of **Fc^+^** in different abiological model solutions at two temperatures (25 °C and 37 °C), cyclic voltammetry and UV-vis spectroscopy were used (Note: a selection of data are reported in Figure 6; see also Figure S1, Supplementary Material, for the complete set of experiments). In pure water at 25 °C, **Fc^+^** is reasonably stable, reaching 81% of the original absorbance in 48 h; at 37 °C, it is slightly less stable, reaching a normalized absorbance value of 76% (initial pH = 7.25; final pH = 6.95).

The presence of salts, in particular, has a major effect on the stability of **Fc^+^** for a long time in a solution. In phosphate-buffered saline (PBS, pH = 7.4), the concentration of **Fc^+^** is halved after approximately 31 h at 25 °C but there was a dramatic drop to 1.8% at 37 °C after 25 h. It is interesting that at the end of the analysis, traces of a white floccose precipitate are visible, suggesting the formation of hydroxido- and phosphato-containing complexes.

An increase in the complexity of the medium results in a further reduction in stability. In RPMI 1640 cell culture medium added with 10% fetal bovine serum (FBS), the UV-vis absorption of **Fc^+^** decreased after 24 h to 15% at 25 °C and to <4% at 37 °C, respectively. In addition, a new peak at 516 nm is observed in these cases. The PAS + AX-2 medium (dicty medium) has an effect at 25 °C that is similar to that observed with RPMI 1640 at 37 °C.

**Figure 7:**
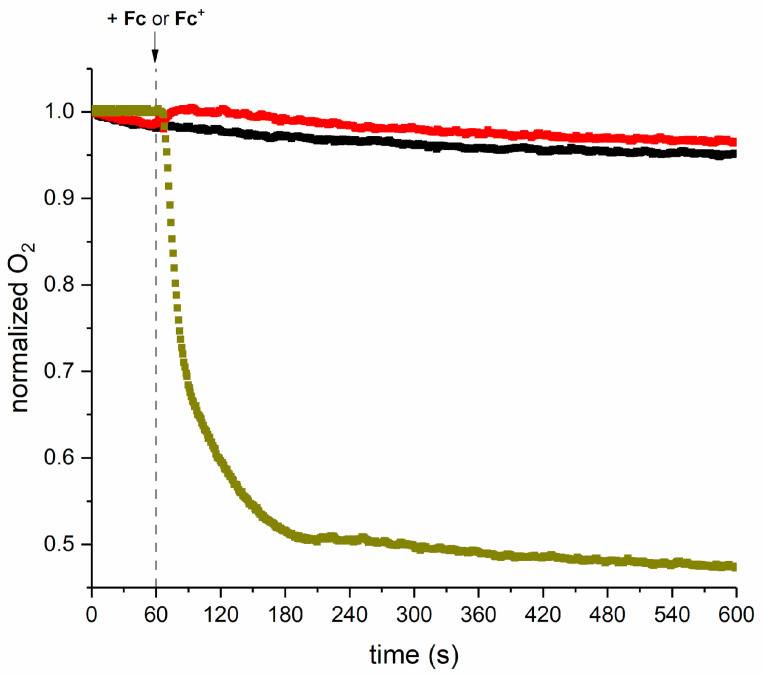
Dissolved oxygen vs. time determined by a Clark-type O_2_ electrode in the absence (black squares, 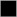) or presence of 2.7 mM concentrations of **Fc** (in PBS + 2.7% *v/v* DMSO; red squares, 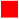) and **Fc^+^** (in PBS; dark yellow, 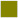).

A Clark-type oxygen electrode was used to evaluate the O_2_ consumption of **Fc** and **Fc^+^** (Figure 7). The cell contained PBS and the signal was stabilized for 60 s. After this time, solid **Fc^+^** or a concentrated solution of **Fc** in DMSO was added (final concentrations 3 mM; in the case of **Fc,** DMSO was 3% *v/v*) and the experiment was continued for 10 h. In the presence of **Fc**, the trend of the normalized O_2_ content was practically overlaid on that of the blank solution. Surprisingly, in the presence of **Fc^+^**, a normalized O_2_ content of 0.5 was reached 120 min after compound addition. However, this is in line with the previous finding that **Fc^+^** is sensitive to O_2_ and its decomposition depends on the solvent and the presence of other reagents, also involving Cp ligands in reactions [57,58,59,60].

**Figure 8:**
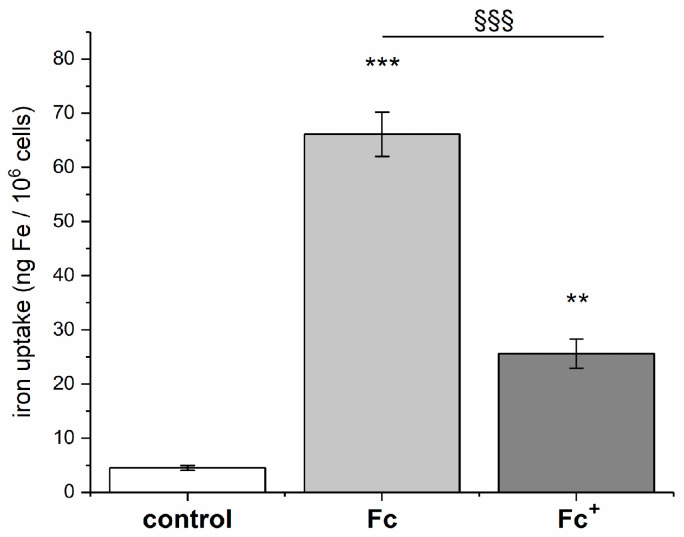
Iron uptake of **Fc** (light grey) and **Fc^+^** (dark grey) in dicty cells treated for 30 min with 100 µM concentrations of the compounds in isotonic buffer (control experiment is in white). Data are the mean ± sd of three independent replicates and were compared using a one-way Anova analysis of the variance-Tukey test. Statistical analysis (**Fc** and **Fc^+^** vs. control): (**) *p* < 0.01; (***) *p* < 0.001; (**Fc** vs. **Fc^+^**): (§§§) *p* < 0.001.

Finally, the cellular uptake of **Fc** and **Fc^+^** was measured in dicty cells after a 30 min treatment with 100 µM concentrations of iron compounds. As expected, **Fc** showed a much higher internalization due to its higher lipophilicity (Figure 8).

### 2.4. Immunocytochemical Staining Revealed a Time-Dependent Activation of Different Cell Death Mechanisms

After confirming that ROS formation is associated with the treatment of **Fc** and **Fc^+^**, the regulators of three types of cell death (i.e., apoptosis, necroptosis, and ferroptosis) were investigated at different time points in MCF-7 breast cancer cells.

Apoptosis is a highly regulated and conserved process during which a cell undergoes self-destruction to eliminate unwanted, superfluous, or DNA-damaged cells. Oxidative stress or alkylating agents promote the formation of a large number of DNA breaks that induce overactivation of the nuclear enzyme Poly(ADP-ribose) polymerase-1 (PARP1). Depending on the degree of injury and cell type, PARP1 activation can lead to apoptosis, representing a marker of such a cell death mechanism.

**Figure 9:**
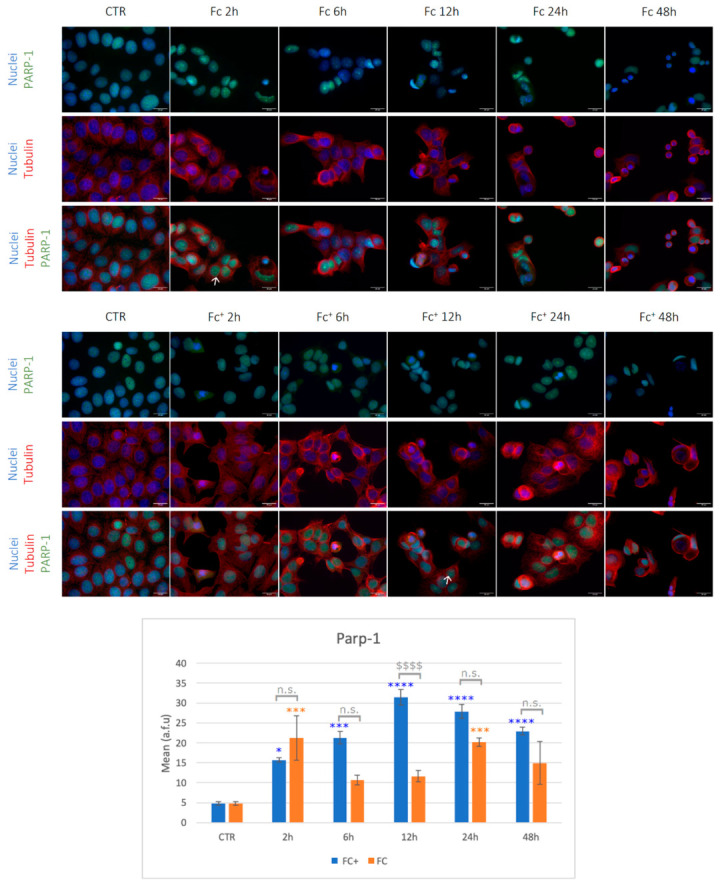
(**Top**) Double immunocytochemical reaction for PARP-1 (green fluorescence) and tubulin (red fluorescence); DNA counterstaining with Hoechst 33258 (blue fluorescence) under control condition and samples treated with **Fc** and **Fc^+^** (200 μM). Bar = 20 μm; magnification: 60×. See the main text for the meaning of the arrows. (**Bottom**) The histogram represents the relative expression of PARP-1. Statistical analysis (one-way Anova): *p* < 0.0001. Statistical significance between * control condition and treated samples; statistical significance between $ **Fc**- and **Fc^+^**-treated samples (two-tailed unpaired *t*-test **Fc^+^** vs. **Fc**); *p*-values: (*) *p* < 0.05; (***) *p* < 0.005; ($$$$), (****) *p* < 0.0001; n.s. = not significant.

As shown in Figure 9, cells showed mild nuclear immunopositivity for PARP-1 (green) under control conditions. In contrast, tubulin (i.e., the protein that polymerizes into long chains that form hollow fibers called microtubules that serve as a skeletal system for the cell) was well structured in thin filaments within the cytoplasm (indicated by an arrow).

After treatments with both **Fc** and **Fc^+^**, cells with apoptotic features (i.e., round-shaped cells with a collapsed cytoskeleton and reduced dimensions) were detected, especially in the longer lasting treatments with both **Fc** and **Fc^+^**. Furthermore, the PARP-1 fluorescent signal was clearly more intense among all conditions tested and was located both in the nucleus and nucleoli (indicated by arrows). Fluorescence analysis revealed statistical differences between the tested conditions: in **Fc**-treated cells, the highest levels of PARP-1 activation were detected after 2 h of exposition compared to controls, whereas in the **Fc^+^**-treated samples, the highest levels of PARP-1 expression were detected after 12 h (indicated by arrows). In particular, the highest levels of PARP-1 were detected after 2 h of CT with **Fc**, which corresponds to the large presence of dead cells during the flow cytometry experiment. Furthermore, after the activation peak in **Fc**-treated samples, the PARP-1 expression gradually decreased, although significantly higher after 24 h. This possibly indicates the presence of apoptosis-resistant cells, as also confirmed by the peaks observed in the longer lasting treatment of the cell cycle analysis and the presence of sub-G1 peaks in the late time points.

Necroptosis is another regulated cell death pathway; it depends on the phosphorylation of mixed lineage kinase (MLKL) by receptor-interacting kinase 1 and 3 (RIP1 and RIP3, respectively). Specifically, RIP1 recruits RIP3, which becomes phosphorylated and active. At this point, the necrosome is formed and RIP3 recruits and phosphorylates MLKL. After phosphorylation, MLKL undergoes conformational changes and translocates to the plasma membrane and the nucleus. This process eventually leads to cell death characterized by permeabilization of the plasma membrane, cell swelling, and loss of cell and organelle integrity. Recently, the importance of necroptosis in cancer has become increasingly appreciated and a better understanding of necroptotic processes could be helpful in creating new strategies to control cancer [61].

**Figure 10:**
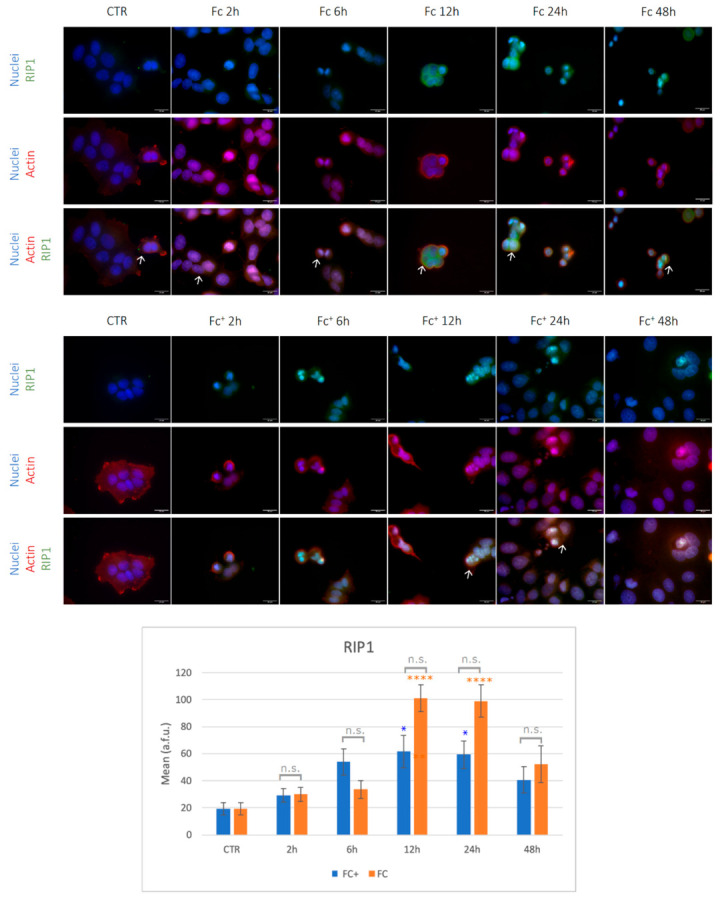
(**Top**) Double immunocytochemical reaction for RIP1 (green fluorescence) and actin (red fluorescence); DNA counterstaining with Hoechst 33258 (blue fluorescence) in control conditions and **Fc**- and **Fc^+^**-treated samples (200 μM). Bar = 20 μm; magnification: 60×. See the main text for the meaning of the arrows. (**Bottom**). The histogram represents the relative expression of RIP1. Statistical analysis (one-way Anova): *p* < 0.0001. Statistical significance between * control condition and treated samples; statistical significance between $ **Fc**- and **Fc^+^**-treated samples (two-tailed unpaired *t*-test **Fc^+^** vs. **Fc**); *p*-values: (*) *p* < 0.05, (****) *p* < 0.0001; n.s. = not significant.

The necroptotic pathway was evaluated by immunocytochemical staining for RIP1 and MLKL. Under control conditions and after 2, 6, and 48 h of CT with the two compounds tested, the RIP1 signal was barely observable and was located primarily in the cytoplasm (Figure 10, indicated by arrows).

Furthermore, under control conditions, the actin cytoskeleton (red fluorescence) was well structured into a thin filament throughout the cytoplasm and collapsed after treatment with iron compounds (Figure 10). However, after 12 and 24 h of CT with both **Fc** and **Fc^+^**, RIP1 was significantly overexpressed compared to the control conditions (see Table S1, Supplementary Material). Furthermore, it was located in both cytoplasmic and nuclear compartments (indicated by arrows), with more intense nuclear staining in round cells. Importantly, RIP1 is significantly overexpressed in **Fc**-treated samples compared to **Fc^+^** samples at both 12 and 24 h time points.

**Figure 11:**
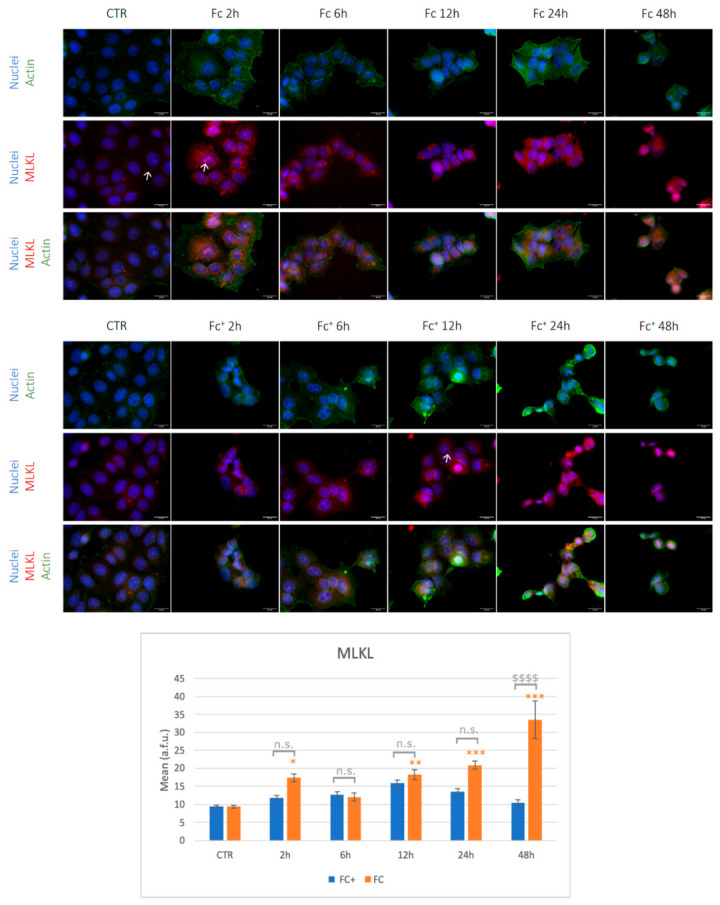
(**Top**) Double immunocytochemical reaction for actin (green fluorescence) and MLKL (red fluorescence); DNA counterstaining with Hoechst 33258 (blue fluorescence) under control conditions and **Fc**- and **Fc^+^**-treated samples (200 μM). Bar = 20 μm; magnification: 60×. See the main text for the meaning of the arrows. (**Bottom**) The histogram represents the relative expression of MLKL. Statistical analysis (one-way Anova): *p* < 0.0001. Statistical significance between * control condition and treated samples; statistical significance between $ **Fc**- and **Fc^+^**-treated samples (two-tailed unpaired *t*-test **Fc^+^** vs. **Fc**); *p*-values: (*) *p* < 0.05; (**) *p* < 0.01; (***) *p* < 0.005; ($$$$); n.s. = not significant.

Considering MLKL expression (Figure 11, see also Table S1, Supplementary Material), its staining was mainly cytoplasmic under control conditions (indicated by arrows). Overall, after exposure to **Fc**, the signal was significantly higher compared to the control condition (with the exception of the 6 h time point which did not reach statistical significance, even if a trend towards overexpression is detected) and was also localized in the nucleoli (indicated by arrows). Moving to **Fc^+^**-treated samples, no significant changes in MLKL expression were detected, even if a trend toward overexpression was observed after 12 h of treatments vs. controls. In particular, this is also the only time point in which the red signals were mainly localized in the cytoplasm and nucleus, whereas in the other conditions tested, MLKL stained primarily the cytoplasm (Figure 11, indicated by arrows).

These data point to an activation of the necroptotic pathway at very early time points after exposure to **Fc** alone, reaching the peak after 48 h. This hypothesis is supported by the stronger activation of RIP1, especially after 12 and 24 h, in **Fc**-exposed cells. On the contrary, the activation of RIP1 but not of MLKL in **Fc^+^**-treated cells suggests a major involvement of the apoptotic pathway rather than the necroptotic one, in accordance with the significantly higher PARP-1 levels previously observed.

**Figure 12:**
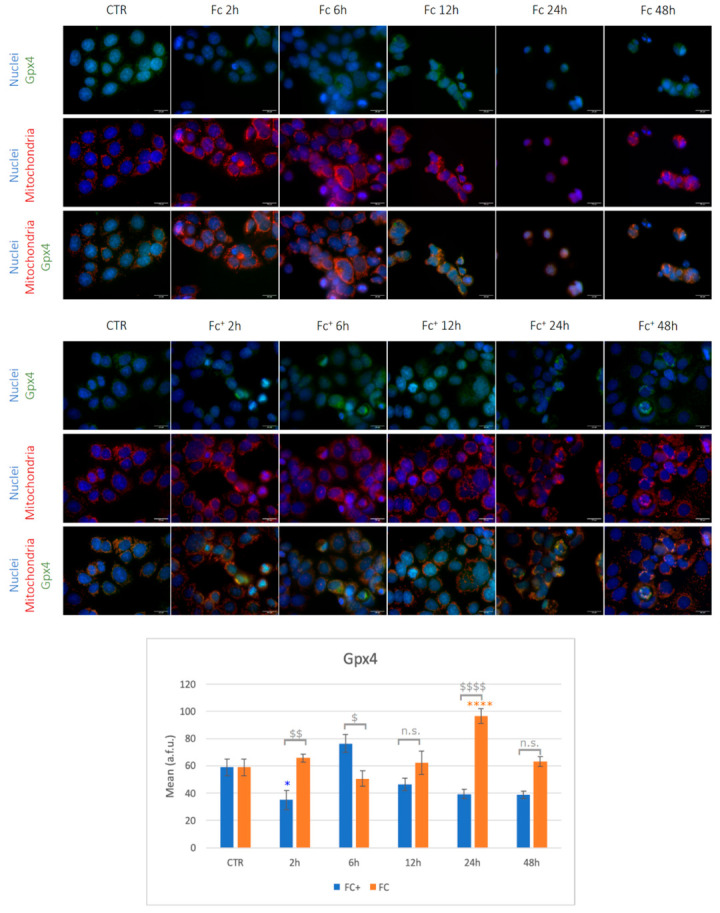
(**Top**) Double immunocytochemical reaction for Gpx4 (green fluorescence) and mitochondria (red fluorescence); DNA counterstaining with Hoechst 33258 (blue fluorescence) in control conditions and **Fc-** and **Fc^+^**-treated samples (200 μM). Bar = 20 μm; magnification: 60×. (**Bottom**) The histogram represents the relative expression of GPX4. Statistical analysis (one-way Anova): *p* < 0.0001. Statistical significance between * control condition and treated samples; statistical significance between $ **Fc-** and **Fc^+^**-treated samples (two-tailed unpaired *t*-test **Fc^+^** vs. **Fc**); *p*-values: ($), (*) *p* < 0.05; ($$) *p* < 0.01; ($$$$), (****) *p* < 0.0001; n.s. = not significant.

As already mentioned in the Introduction, ferroptosis depends on the accumulation of excessive divalent iron in cells, which catalyzes the lipid peroxidation of unsaturated fatty acids that are highly expressed on the cell membrane. The selenoenzyme glutathione peroxidase 4 (Gpx4) belongs to the glutathione peroxidase family and is the only member of the group that showed the ability to scavenge lipid ROS products. For this reason, Gpx4 exerts a protective role against cell death, and, at the same time, its expression can be evaluated as a selective indicator of ferroptosis. Under control conditions, cells showed light immunopositivity for the investigated marker (Figure 12, see also Table S1, Supplementary Material). After treatments with **Fc**, a stronger Gpx4 signal was detected at the different time points, reaching statistical significance after 24 h of treatment with respect to the control conditions, except for a slight decrease in Gpx4 expression after 6 h. Interestingly, cells treated with **Fc^+^** followed an opposite trend: a general downregulation of Gpx4 was detected, although statistically significant only after 2 h of treatments. On the contrary, after 6 h of CT, minor overexpression was observed. Finally, Gpx4 was significantly higher in 2, 6, and 24 h **Fc**-treated samples, while after 6 h, its expression was higher in **Fc^+^**. Overall, these data suggest a rapid activation of ferroptosis in **Fc^+^**-treated samples only, probably because of its intrinsic instability which results in a faster degradation compared to that of **Fc**. On the other hand, **Fc** failed as a ferroptosis inducer but efficiently activated necroptotic and apoptotic pathways, especially after longer exposures.

### 2.5. Ultrastructural Analysis

**Figure 13:**
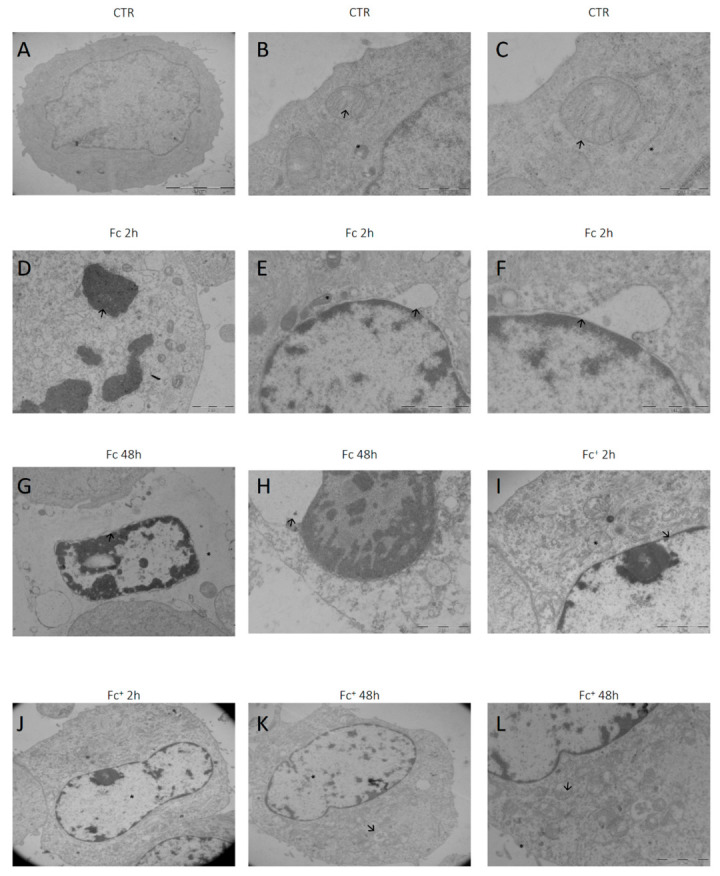
Ultrastructural analysis by TEM. (**A**–**C**) MCF-7 cells under control (CTR) conditions. (**B**,**C**) Healthy mitochondria with well-organized cristae, indicated by arrows. (**D**,**F**) Cells after 2 h of continuous treatment with **Fc** 200 μM. (**D**) Apoptotic and (**E**) necroptotic cells with altered mitochondria (indicated by an asterisk in **E**) and a detached perinuclear space, highlighted by arrows in (**E**,**F**). (**G**,**H**) Cells exposed for 48 h to **Fc** 200 μM. Advanced necroptotic phase showing an electron-lucent cytoplasm (indicated by an asterisk in **G**), a more condensed chromatin, and an extreme enlargement of the perinuclear space (arrows). (**I**,**J**) Cells treated for 2 h with **Fc^+^** 200 μM. No enlargement of the perinuclear space was observed (arrow in **I**). Examples of altered mitochondria with impaired cristae structure (highlighted by asterisk in **I**) and ferroptotic cell with a decondensed chromatin (pointed by asterisk in **J**). (**K**,**L**) Cells after 48 h of CT with **Fc^+^** 200 μM. Examples of late ferroptosis (**K**,**L**) characterized by decondensed chromatin (asterisk in **K**) and severely impaired mitochondria (highlighted by arrows in **K**,**L**).

Finally, to confirm the activation of different cell death mechanisms, the morphological changes in MCF-7 cells were analyzed by transmission electron microscopy after 2 h and 48 h of CT with **Fc** and **Fc^+^**. The untreated cells (Figure 13A) were characterized by the nucleus in the central position, decondensed chromatin, and a well-organized Golgi apparatus in the perinuclear zone (Figure 13C, indicated by an arrow). Furthermore, in the cytoplasm, medium-sized mitochondria with regular cristae structures (Figure 13B,C, indicated by an asterisk in Figure 13B) and sporadic lysosomes with basal autophagic activity were observed (Figure 13C, indicated by an asterisk). After 2 h of CT with **Fc**, cells showed an apoptotic morphology, characterized by condensed chromatin (Figure 13D, arrow), a ruptured nuclear envelope, and cytosol degradation. Furthermore, several cells exhibited early necroptotic features (i.e., an electron-lucent cytoplasm, the detachment of the perinuclear space, and altered mitochondria, see Figure 13E,F, indicated by an arrow and asterisk, respectively). Progressively, necroptotic cells appeared with more condensed DNA, a completely disrupted cytoplasm, and a massive enlargement of the perinuclear space, after 48 h of CT with **Fc** (Figure 13F, arrows and asterisks, respectively, and Figure 13G). On the contrary, no necroptotic cells were observed either after treatment with **Fc^+^**. However, after 2 h of treatment with the positive-charged compound, the cells showed severely altered mitochondria, with an electron-lucent matrix and altered cristae structures (Figure 13H,I, indicated by an asterisk and arrow, respectively). Furthermore, the same cells also exhibited extremely decondensed chromatin with no alterations involving the perinuclear space, indicating the activation of ferroptosis. After 48 h of CT with **Fc^+^**, late stage ferroptotic cells were identified: chromatin appeared completely relaxed and electron-lucent (Figure 13K, asterisk), whereas the cytoplasm was enriched in altered mitochondria and vesicles containing damaged organelles (Figure 13K,L, arrows). Finally, the cell membranes began to lose their integrity (Figure 13L, asterisk).

## 3. Materials and Methods

### 3.1. Chemistry

Ferrocene (**Fc**) and other chemicals were purchased from Sigma-Aldrich-Merck (Milan, Italy), except where otherwise indicated, and were used as received. Ferrocenium tetrafluoroborate ([**Fc^+^**][BF_4_]) was prepared according to established procedures [62]. Briefly, **Fc** (1 mmol, 186 mg) was oxidized with *p*-benzoquinone (2 mmol, 216 mg) in the presence of tetrafluoroboric acid (4 mmol, 352 mg, commercially available as a 48% *w*/*w* solution in H_2_O) in 30 mL of diethyl ether. The resulting blue precipitate of [**Fc^+^**][BF_4_] was filtered and thoroughly washed with diethyl ether.

UV-vis spectra were recorded on a JASCO V-550 spectrophotometer equipped with a Peltier thermostated cell holder as a temperature control system. The spectra of 1 mM solutions of **Fc^+^** in solution were recorded in the 300–800 nm range up to 48 h.

Electrochemical measurements were performed using an Autolab PGSTAT12 electrochemical analyzer (Eco Chemie, Utrecht, The Netherlands) interfaced with a personal computer running GPES 4.9 electrochemical software. A standard three-electrode glass cell was designed to allow the tip of the reference electrode (Ag/AgCl, 3 M KCl) to closely approach the working electrode (a glassy carbon, GC, disk, diameter 0.1 cm, sealed in epoxy resin) and the auxiliary electrode (a Pt wire). The working electrode was polished with alumina, rinsed with distilled water, and dried. This process yielded an almost completely reproducible surface for all experiments. All measurements were carried out under N_2_ in the medium under investigation; [**Fc^+^**] was 0.5 mM. The temperature of the solution was kept constant by circulation of a thermostated water/ethanol mixture through a jacketed cell. All peak currents were measured at the 0.2 V s^−1^ scan rate.

Oxygen consumption was followed using a standard Clark-type O_2_ electrode (Hansatech Instruments, Norfolk, UK). The signal was recorded throughout the duration of the experiment at 1 s intervals using Oxygraph software version 1.02 (Hansatech Instruments, Norfolk, UK).

### 3.2. Cell Culture and Treatments

Triple negative human breast cancer cells MCF-7 (ATCC HBT-22) (ATCC, Rockville, MD, USA) were cultured in Minimum Essential Medium (MEM) (Euroclone, Milan, Italy) supplemented with 1% penicillin/streptomycin and 10% fetal bovine serum (FBS) and kept at 37 °C in a humidified atmosphere (95% air 5% CO_2_). Forty-eight hours before experiments, cells were seeded on glass coverslips (200,000 cells) for fluorescence microscopy or grown in 75 cm^2^ plastic flasks for flow cytometric and ultrastructural analysis at transmission electron microscope. Cells were treated for 12 and 48 h with the compounds under investigation, then flow cytometry, ultrastructural analysis, and immunocytochemical analysis were performed. A concentration of 200 μM for **Fc** and [**Fc^+^**][BF_4_] was chosen after reviewing the literature [23,24].

The *Dictyostelium discoideum* (*D. discoideum*, dicty) AX-2 strain was grown in axenic medium (AX-2 medium; 14.3 g L^−1^ bacteriological peptone; 7.15 g L^−1^ yeast extract; 18 g L^−1^ maltose; 0.419 g L^−1^ Na_2_HPO_4_; 0.486 g L^−1^ KH_2_PO_4_, pH 6.6) in the presence of 10 μg mL^−1^ tetracycline in a Gallenkamp orbital incubator (Sanyo Gallenkamp Plc., Loughborough, UK) at 180 rpm and at 21 °C [63]. Cells at the late logarithmic phase (2–4 × 10^6^ cells mL^−1^) were stored overnight at 4 °C to synchronize the amoebae cell cycles. The cells were transferred in Page’s Amoeba Solution (PAS; 2 mM NaCl, 15 μM MgSO_4_, 2.5 μM CaCl_2_, 1 mM Na_2_HPO_4_, 1 mM KH_2_PO_4_, pH 6.4) supplemented with 25% AX-2 and 50 μM CaCl_2_ for 6 h in a Gallenkamp orbital incubator as described above. Control and treated amoebae were grown under the treated cells’ same conditions, but the medium was added with **Fc** and **Fc^+^**. Two stock solutions were prepared by dissolving **Fc^+^** in water and **Fc** in DMSO; the solutions were added at the desired concentration to dicty. To exclude any effects of the DMSO vehicle, the same solvent volume was added to the control cells and the **Fc^+^**-exposed cells at the same dilution.

### 3.3. Determination of Iron Uptake in Dicty Cells

For iron accumulation measurements, dicty cells were washed twice with PAS, transferred to a borosilicate glass tube, and centrifuged at 500× *g* for 10 min at room temperature (RT). The supernatant was carefully removed by aspiration, while approximately 200 μL of the supernatant was left to limit cell loss. Cellular pellets were stored at −80 °C until mineralization. The determination of iron content was performed by ICP-MS (Thermo Optek X Series 2). Mineralization was carried out by adding 70% *w*/*w* HNO_3_ to each sample (after defrosting), followed by incubation for 1 h at 60 °C in an ultrasonic bath. Before ICP-MS measurement, HNO_3_ was diluted to a final concentration of 1%. Instrument settings were optimized to produce the highest iron sensitivity and the isotopes of Fe and In isotopes (used as an internal standard) were quantified at *m/z* 56 and 115, respectively. The iron content was normalized to 10^6^ dicty cells and expressed as ng Fe per 10^−6^ cells.

### 3.4. Cell Cycle Analysis

The cells were rapidly washed in phosphate-buffered saline (PBS), permeabilized in 70% ethanol for 10 min, treated with RNase A 100 U mL^−1^, and then stained at RT with 50 μg mL^−1^ propidium iodide (PI) (Sigma-Aldrich, Milan, Italy) 1 h before flow cytometric analysis. PI red fluorescence was detected with a 610 nm long-pass emission filter. At least 10,000 cells per sample were measured to obtain the distribution among the different phases of the cell cycle and the percentage of dead cells. Data were collected using Cytometer BD FACS Lyric (Becton Dickinson, Franklin Lakes, NJ, USA) and analyzed with FlowJo software (v.10.9, Becton, Dickinson & Company, Franklin Lakes, NJ, USA).

### 3.5. Detection of Intracellular ROS Production in Dicty Cells

For evaluation of intracellular ROS production, 40 μL of amoebae culture was pipetted onto microscope slides in a wet chamber at room temperature where cells adhered to glass in 10 min. The slides were washed with PAS solution to eliminate highly fluorescent culture medium. The cells were treated for 30 min at room temperature with PAS containing 2′,7′-Dichlorodihydrofluorescein diacetate (H_2_DCF-DA). The excess reagent was washed with PAS medium. Differences in fluorescence between treated and control samples were evaluated by an inverted fluorescence microscope equipped with a FITC filter. The images obtained were analyzed using an image analysis system (Scion Image freeware v 1.62).

### 3.6. Immunocytochemical Reactions: Fluorescence Microscopy Evaluation

Control and treated cells grown on coverslips were fixed with 4% formalin for 20 min and post-fixed with 70% ethanol at −20 °C for at least 24 h. The samples were rehydrated for 10 min in PBS and then unspecific sites were blocked using PBS supplemented with 4% BSA and 0.2% Tween for 15 min at RT. Subsequently, cells were immunolabeled with primary antibodies diluted in 0.2% PBS-Tween for 1 h at RT in a dark moist chamber. After 3 washes in PBS of 5 min each, coverslips were incubated with secondary antibodies in 0.2% PBS-Tween (1:200, Alexa Fluor, Molecular Probes, Eugene, OR, USA, Thermo Fisher Scientific, Waltham, MA, USA) for 45 min. At the end of the incubation and after other washings in PBS, sections were counterstained for DNA with 0.1 μg mL^−1^. Hoechst 33258 (Sigma-Aldrich, Milan, Italy), washed with PBS, and mounted in a drop of Mowiol (Calbiochem, Inalco, Italy) for fluorescence microscopy analysis. Primary and secondary antibodies used for immunocytochemical reactions at the fluorescence microscope are reported in Table 1.

An Olympus BX51 microscope equipped with a 100 W mercury lamp was used under the following conditions: 330–385 nm excitation filter (excf), 400 nm dichroic mirror (dm), and 420 nm barrier filter (bf) for Hoechst 33258; 450–480 nm excf, 500 nm dm, and 515 nm bf for the fluorescence of Alexa 488; 540 nm excf, 580 nm dm, and 620 nm bf for Alexa 594. The images were recorded with an Olympus MagnaFire camera system and processed with Olympus Cell F software version 3.1 (Olympus Italia Srl, Segrate, MI, Italy). To make the fluorescence intensity comparable, during image acquisition the exposure time to detect every single fluorescence was selected based on the control sample and then kept constant for the respective experimental conditions, thus avoiding the insertion of any variables in the analysis. The fluorescence intensity of the proteins of interest was analyzed using ImageJ software version 1.54f [64] on eleven quadrants for each sample under analysis. The mean fluorescence intensity of the background noise was subtracted for each field of view.

### 3.7. Transmission Electron Microscopy (TEM)

Control and treated cells were harvested by mild trypsinization (0.25% trypsin in PBS containing 0.05% EDTA) and centrifuged at 800 rpm for 5 min in fresh tubes. The samples were immediately fixed with 2.5% glutaraldehyde in culture medium (2 h at RT) and washed several times with PBS. The samples were stained with 1% OsO_4_ for 2 h at RT and washed in distilled water. Cell pellets were pre-embedded in 2% agar, dehydrated with increasing concentrations of acetone (30%, 50%, 70%, 90%, and 100%), and finally embedded in epoxy resin EM-bed812 (Electron Microscopy Sciences, Hatfield, PA, USA) and polymerized at 60 °C for 48 h. Ultrathin sections (60–80 nm) were cut on a Reichert OM-U3 ultramicrotome, collected on nickel grids, and stained with uranyl acetate and lead citrate. Finally, sections were observed under a JEM 1200 EX II electron microscope (JEOL, Peabody, MA, USA) equipped with a MegaView G2 CCD camera (Olympus OSIS, Tokyo, Japan) operating at 100 kV.

### 3.8. Statistical Analysis

Data were expressed as mean ± SEM (standard error of the mean). Data differences were analyzed for statistical significance using one-way ANOVA and post hoc Bonferroni test using GraphPad Prism version 8.4 for Windows (GraphPad Software, http://www.graphpad.com (accessed on 1 September 2023)). The data differences between the treatment with **Fc** and **Fc^+^** among the tested time points were analyzed using an unpaired two-tailed *t*-test. *p*-values < 0.05 were considered statistically significant.

## 4. Conclusions

**Figure 14:**
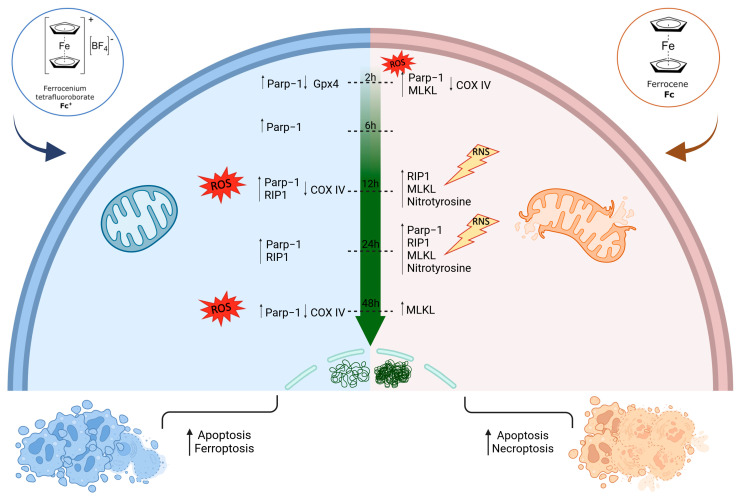
Both **Fc** and even more **Fc^+^** exposure resulted in the apoptotic cell death of MCF-7 cancer cells at the tested time points. Furthermore, the intrinsic instability of **Fc^+^** caused the release of the central iron atom and the early activation of ferroptosis, followed by an increase in oxidative stress. Ferroptotic cells showed enlarged mitochondria with abnormal cristae and extreme decondensed chromatin. On the other hand, the more stable **Fc** led to the activation of the necroptotic cell death mechanism, especially after longer-lasting treatments, because of the immediate production of ROS and the consequent RNS accumulation. These cells appeared with altered mitochondria and more condensed chromatin. The image was made with BioRender.com (accessed on 1 September 2023).

In the present work, it has been shown that both **Fc** and **Fc^+^** may trigger alternative mechanisms of cell death and cytotoxicity after different long-term exposures due to the different stability of the tested compounds in biological media. Figure 14 summarizes the main observations obtained in the present work.

Variations in COXIV levels in MCF-7 breast cancer cells treated with the compounds under investigation point to further confirmation of ROS formation and accumulation. Moreover, it is already well established that reduced levels of COXIV expression have toxic effects on mitochondrial metabolism, which in turn leads to the activation of apoptosis [65,66,67]. In addition, different morphological changes were observed in MCF-7 cells after **Fc** and **Fc^+^** treatment (i.e., cells grouped together, elongated and clustered mitochondria, a collapsed cytoskeleton, a decreased cell volume) and are consistent with the effect of ROS. Tests performed on the social amoeba and model organism dicty confirmed the ROS accumulation, in particular, in the case of **Fc^+^**.

Furthermore, the activation of apoptosis in MCF-7 cells as a consequence of ROS production is confirmed by the increase in PARP-1 levels, especially after **Fc^+^** treatments. Furthermore, higher ROS levels may be the result of iron accumulation once the compound is decomposed externally to cells (**Fc^+^**) or has been metabolized by cells (**Fc**), leading to lipid peroxidation and ferroptotic cell death. Gpx4 was found to be significantly downregulated after 2 h of **Fc^+^** treatments, indicating the ability of this compound to exert cytotoxic effects in a very short time and, specifically, to induce ferroptosis. Even in longer-lasting treatments, Gpx4 showed a trend towards a lower expression after exposure to **Fc^+^**, apart from the 6 h CT time point. This transient up-regulation is probably an attempt by cells to mitigate the toxic effects triggered by the compound. This result is very promising, in our view, as it is well established that targeting ferroptosis could be an extremely valuable option in cancer treatment. Furthermore, excess iron that is not used or stored can be exported across the cell membrane through ferroportin, an iron efflux pump that works in conjunction with hephaestin or ceruloplasmin to maintain cellular iron homeostasis. Importantly, ferroportin is intensely suppressed in many types of cancer, including breast cancer [68], meaning that cancer cells will be more susceptible to iron overload, a major ferroptotic trigger.

A significant time delay in the formation of iron species capable of inducing ROS formation from **Fc** and **Fc^+^** was observed when studying necroptosis. RIP1 and, more importantly, MLKL were up-regulated after 2, 24, and 48 h of CT with **Fc**, while higher levels after **Fc^+^** treatment of RIP1 only are detectable after 12 and 24 h of exposure.

Finally, the ultrastructural analysis supports all the results presented here: ROS production severely affects mitochondrial structures, especially after **Fc^+^** treatments, and results in the activation of apoptosis. Furthermore, ferroptotic cell death occurs after 2 h of CT with **Fc^+^** but not after **Fc**, probably due to the greater instability of ferrocenium and its consequent degradation outside the cells. At this point, the released Fe^3+^ species immediately enhance the ROS production, which in turn react with the polyunsaturated fatty acids of the cell membrane, resulting in the ferroptotic cell death. On the other hand, the more stable **Fc** shows its cytotoxic effect by activating necroptosis after a longer-lasting treatment. The differences observed in terms of cell death mechanisms and timing may be due to rapid interconversion between the two oxidative forms of internalized iron species (from Fe^2+^ to Fe^3+^ and vice versa).

New studies have shown that Gpx4 can cause other types of cell death, including apoptosis [69], autophagy [70], necroptosis [71], and pyroptosis [72], indicating that there is a potential interdependence between ferroptosis and other programmed cell death. In this view, it may be interesting to analyze mitophagy [73] and other mitochondrial functions, such as Opa1, PINK1, Parkin, and Aco-2, to deeply characterize their disfunction in relation to ROS production and alternative cell death mechanisms. A potential limitation of the study is that the iron-responsive elements have not been investigated in this paper.

In conclusion, the triggering of different RCDs makes ferrocene a multitarget moiety and a potential agent that could overcome the shortcomings of traditional therapies mediated by the apoptotic pathway. One could speculate on its application not as much as standalone anticancer chemotherapeutics but likely as part of a combination therapy or a nanomedicine engineered for the co-delivering of multiple payloads having different targets.

## Figures and Tables

**Table 1:** Primary and secondary antibodies used for immunofluorescence reactions.

Marker	Primary Antibody	Dilution	Secondary Antibody
GPX4	Rabbit polyclonal anti-glutathione peroxidase 4 (GPX4) (Abcam, Cambridge, UK)	1:400	Alexa 488-conjugated anti-rabbit antibody (Molecular Probes, Eugene, OR, USA)
RIP1	Rabbit polyclonal anti-RIP1 (Santa Cruz Biotechnology, Dallas, TX, USA)	1:200	Alexa 488-conjugated anti-rabbit antibody (Molecular Probes, Eugene, OR, USA)
MLKL	Mouse monoclonal anti-MLKL antibody, clone 3H1 (Sigma-Aldrich, Milan, Italy)	1:200	Alexa 594-conjugated anti-mouse antibody (Molecular Probes, Eugene, OR, USA)
PARP-1	Rabbit polyclonal anti-PARP-1 (Cell Signaling Technology, Danvers, MA, USA)	1:200	Alexa 488-conjugated anti-rabbit antibody (Molecular Probes, Eugene, OR, USA)
COXIV	Mouse polyclonal anti-nitrotyrosine antibody (Cell Signaling Technology, Danvers, MA, USA)	1:200	Alexa 594-conjugated anti-mouse antibody (Molecular Probes, Eugene, OR, USA)
Nitrotyrosine	Mouse monoclonal anti-nitrotyrosine antibody (Santa Cruz Biotechnology, Dallas, TX, USA)	1:200	Alexa 594-conjugated anti-mouse antibody (Molecular Probes, Eugene, OR, USA)
β-Actin	Rabbit polyclonal anti-beta actin (GeneText, Irvine, CA, USA)	1:200	Alexa 488-conjugated anti-rabbit antibody (Molecular Probes, Eugene, OR, USA)
α-tubulin	Monoclonal mouse anti-α-tubulin (Invitrogen, Waltham, MA, USA)	1:1000	Alexa 594-conjugated anti-mouse antibody (Molecular Probes, Eugene, OR, USA)
Mitochondria	Human autoimmune serum recognizing the 70 kDa E2 subunit of the pyruvate dehydrogenase complex b	1:400	Alexa 594-conjugated anti-human antibody (Molecular Probes, Eugene, OR, USA)

## Data Availability

No research data will be provided beyond those present in the text and the Supplementary Materials.

## Supplementary Materials

The following supporting information can be downloaded at: https://www.mdpi.com/article/10.3390/molecules28186469/s1, Figure S1: Stability in solution vs. aging time in different model solutions, at 25 °C and 37 °C; Table S1: Summary of the semi-quantitative analysis of immunofluorescence reactions.
