# Supplementary material for: Effects of Ferrocene and Ferrocenium on MCF-7 Breast Cancer Cells and Interconnection with Regulated Cell Death Pathways
Source: Molecules. 2023 Sep 6;28(18):6469. doi: 10.3390/molecules28186469 (PMC10537025; doi:10.3390/molecules28186469)
Supplement: Supplementary file 1 [file molecules-28-06469-s001.zip › molecules-2540285-supplementary.pdf]

Effects of Ferrocene and Ferrocenium on MCF-7 Breast Cancer Cells and Interconnection with Regulated Cell Death Pathways

Cristina Favaron [1], Elisabetta Gabano [2], Ilaria Zanellato [3], Ludovica Gaiaschi [1], Claudio Casali [1], Maria Grazia Bottone [1,*], and Mauro Ravera [3,*]

[1] Department of Biology and Biotechnology "L. Spallanzani", University of Pavia, Via Ferrata 9, 27100 Pavia, Italy
[2] Department of Sustainable Development and Ecological Transition, University of Piemonte Orientale, Piazza S. Eusebio 5, 13100 Vercelli, Italy
[3] Department of Sciences and Technological Innovation, University of Piemonte Orientale, Viale Teresa Michel 11, 15121 Alessandria, Italy
* Correspondence: mariagrazia.bottone@unipv.it (M. G. B.); mauro.ravera@uniupo.it (M. R.)

SUPPLEMENTARY MATERIAL

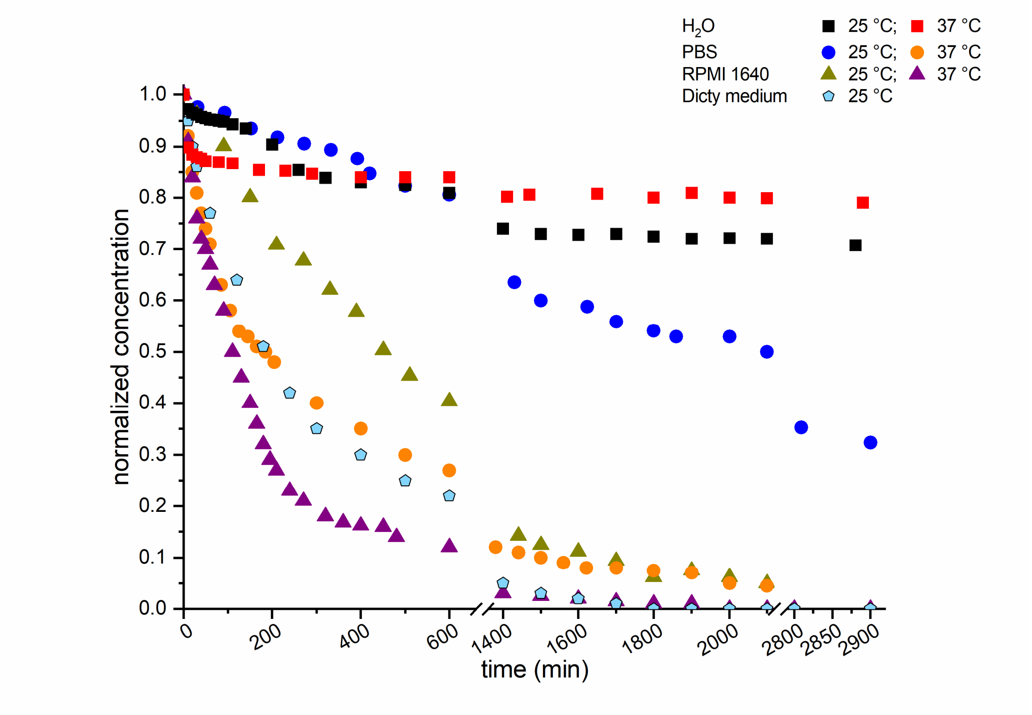

Figure S1. Stability in solution vs. aging time in different model solutions, at 25 °C and 37 °C. Data were obtained by using cyclic voltammetry (peak current, i_p) or UV-vis spectroscopy (absorption A at λ_{max} around 618 nm, depending on the solution). To compare the results, concentrations were normalized against the i_p or A values at time zero.

Table S1. Summary of the semi-quantitative analysis of immunofluorescence reactions. Mean fluorescence intensity (Mean F.I); standard error of the mean (S.E.M); p-value respect to control conditions (one-way Anova; p-value vs. control); two-tailed unpaired t-test **Fc** vs. **Fc$^+$** treatments (t-test **Fc** vs. **Fc$^+$**).

		Fc			Fc$^+$			
		Mean F.I	S.E.M	p-value (vs. CTR)	Mean F.I	S.E.M	p-value (vs. CTR)	t-test (Fc vs. Fc$^+$)
COXIV	CTR	42.538	2.219		42.538	2.219		
	2h	29.389	1.439	0.0006	43.612	4.258	>0.9999	0.0049
	6h	38.512	2.192	>0.9999	37.011	2.198	0.8164	0.634
	12h	35.18	1.162	0.2112	30.637	1.36	0.0035	0.0195
	24h	50.797	2.973	0.0988	40.637	1.322	>0.9999	0.0054
	48h	34.888	2.268	0.1662	30.478	0.738	0.0021	0.0793
Nitrotyrosine	CTR	30.858	2.435		30.858	2.435		
	2h	38.174	5.19	>0.9999	36.16	4.433	>0.9999	0.771
	6h	46.023	3.785	0.6015	34.659	4.474	>0.9999	0.0667
	12h	54.674	6.955	0.0352	33.249	5.401	>0.9999	0.0245
	24h	60.162	7.497	0.0038	27.006	4.902	>0.9999	0.0014
	48h	51.12	9.654	0.1256	31.231	3.579	>0.9999	0.0677
PARP1	CTR	4.84	0.465		4.84	0.465		
	2h	21.223	5.588	0.0002	15.684	0.496	0.0398	0.3353
	6h	10.67	1.132	>0.9999	21.225	1.587	0.0002	<0.0001
	12h	11.646	1.381	0.6756	31.427	1.994	<0.0001	<0.0001
	24h	20.17	1.098	0.0006	27.898	1.785	<0.0001	0.0015
	48h	14.932	5.298	0.0722	22.888	0.958	<0.0001	0.1551
RIP1	CTR	19.477	4.495		19.477	4.495		
	2h	29.921	5.035	>0.9999	29.199	5.124	>0.9999	0.9209
	6h	33.563	6.67	>0.9999	53.861	9.702	0.1146	0.1001
	12h	101.19	9.94	<0.0001	61.56	12.099	0.0212	0.0199
	24h	98.952	11.859	<0.0001	59.36	10.224	0.0352	0.02
	48h	52.323	13.623	0.156	40.734	9.852	>0.9999	0.4985
MLKL	CTR	9.383	0.46		9.383	0.46		
	2h	17.363	1.05	0.0264	11.876	0.613	>0.9999	0.0002
	6h	12.038	1.132	>0.9999	12.734	0.747	>0.9999	0.6134
	12h	18.283	1.381	0.0085	15.901	0.794	0.1342	0.1504
	24h	20.814	1.098	0.0002	13.506	0.849	>0.9999	<0.0001
	48h	33.484	5.298	<0.0001	10.428	0.968	>0.9999	0.0004
Gpx4	CTR	58.844	6.154		58.844	6.154		
	2h	65.672	2.873	>0.9999	34.88	6.793	0.0239	0.0005
	6h	50.627	5.797	>0.9999	76.417	6.703	0.2456	0.0087
	12h	62.314	8.669	>0.9999	46.429	4.388	>0.9999	0.1177
	24h	96.615	5.397	<0.0001	39.176	3.381	0.121	<0.0001
	48h	63.275	3.66	>0.9999	38.566	2.768	0.0975	<0.0001
